# Retraction: Enriched Environment Prevents Hypobaric Hypoxia Induced Memory Impairment and Neurodegeneration: Role of BDNF/PI3K/GSK3β Pathway Coupled with CREB Activation

**DOI:** 10.1371/journal.pone.0317531

**Published:** 2025-01-09

**Authors:** The PLOS ONE Editors

Following the publication of this article [1], concerns were raised regarding results presented in Figs 2 and 5. Specifically,

- In Fig 2, the following panels appear to partially overlap:
  ○ The Fluoro Jade NORMOXIA panel and the Fluoro Jade NORMOXIA + EE panel.
  ○ The Cresyl Violet NORMOXIA panel and the Cresyl Violet NORMOXIA + EE panel.
- There appear to be vertical discontinuities between lanes 1 and 2, and lanes 4 and 5 in the p-GSK-3β panel in Fig 5A.

In response to queries about these experiments, the first author stated that the NORMOXIA + EE panels in Fig 2 are incorrect. They also stated that all bands in Fig 5 are from the same blot but that the underlying data for Fig 5 are no longer available.

The first author provided multiple versions of the underlying data for the charts in Fig 2. These data were reviewed by PLOS and an independent member of the *PLOS ONE* Editorial Board. Upon editorial review, PLOS observed discrepancies in these data and statistics that question the integrity and reliability of Fig 2 as published. In addition, the independent board member noted that it is unclear which pairs in the two-way ANOVA analysis are significant.

In light of the above concerns with Fig 2 that question the integrity and reliability of the reported results and conclusions, the *PLOS ONE* Editors retract this article.

VJ responded but expressed neither agreement nor disagreement with the editorial decision. IB, DP, and GI either did not respond directly or could not be reached.

After the publication of [1], reference 33 was retracted [2–3].
